# Programmed Cell Death in Urological Cancers: Orchestrating the Immune Microenvironment and Immunotherapy

**DOI:** 10.32604/or.2026.087565

**Published:** 2026-09-14

**Authors:** Zhenyang Ye, Jinyang Luo, Ying Zhang, Longhua Lu, Min Lei, Shi Deng

**Affiliations:** 1 Department of Urology, West China Hospital, West China Xiamen Hospital, Sichuan University, Xiamen, China; 2 Department of Urology, West China Hospital, Sichuan University, Chengdu, China

**Keywords:** Programmed cell death, urological cancers, tumor immune microenvironment, immunotherapy, ferroptosis, autophagy

## Abstract

Programmed cell death regulates the tumor immune microenvironment. A comprehensive synthesis of how multiple programmed cell death pathways collectively orchestrate the remodeling of the urological immune landscape is currently lacking. This review summarizes and discusses how diverse programmed cell death modes, including ferroptosis, pyroptosis, autophagy, PANoptosis, necroptosis and cuproptosis, regulate immune evasion or activation in a context-dependent manner. Current preclinical evidence suggests that necroptosis, pyroptosis, and cuproptosis may enhance anti-tumor immunity by facilitating the release of damage-associated molecular patterns and increasing the infiltration of functional CD8^+^ T cells and dendritic cells, thereby potentially improving responses to immunotherapy. At the same time, several programmed cell death pathways display exhibit pronounced context dependence. In renal cell carcinoma, ferroptosis exhibited a functional contradiction: while its induction directly eliminated cancer cells, the resulting lipid peroxidation could simultaneously impair the survival and metabolic fitness of infiltrating immune cells. This dualistic effect necessitated precise, cell-type-specific strategies to ensure that ferroptosis-mediated tumor suppression did not undermine the anti-tumor immune response. Similarly, autophagy in tumor cells facilitated immune evasion via the selective degradation of major histocompatibility complex class I (MHC-I) and stabilizing programmed death-ligand 1, while it also improved the cytotoxic function and cellular longevity of natural killer cells. Consequently, future drug development should consider cell-type-specific modulation to address these contradictory effects across different cells and avoid unintended immunosuppression. Emerging evidence on PANoptosis, which integrates multiple programmed cell death pathways into a synergistic framework, may provide a useful direction for investigating immune therapeutic resistance. Ultimately, targeting the intricate landscape of programmed cell death may inform strategies for improving cancer treatment and urological immunotherapy, but stronger translational evidence is still needed.

## 1 Introduction

Urological cancers, primarily comprising prostate cancer (PCa), urothelial carcinoma (UC, with a predominant focus on bladder cancer (BC)), and renal cell carcinoma (RCC), impose a substantial global health burden [1,2]. Recent epidemiological studies indicated a steadily increasing incidence of these malignancies, further straining global healthcare systems [2,3]. Despite advances in surgery and systemic treatment, outcomes for patients with advanced or metastatic disease remain poor, and resistance to standard-of-care therapy frequently develops [4,5]. RCC is characterized by a distinct metabolic profile and a highly vascular phenotype, whereas PCa frequently presents as an immunologically “cold” tumor with a suppressive architecture that hinders T-cell infiltration [4,6]. UC often exhibits high recurrence rates and heavy molecular heterogeneity, necessitating long-term surveillance and intensified treatment strategies [7,8]. Across these malignancies, a key challenge is the transition from localized disease to systemic progression, which underscores the need for more effective therapeutic approaches. Immune checkpoint inhibitors (ICIs), particularly those targeting programmed cell death protein 1/programmed death-ligand 1 (PD-1/PD-L1) and cytotoxic T-lymphocyte-associated protein 4 (CTLA-4), have reshaped treatment paradigms in selected patients with urological cancer [9,10]. However, clinical efficacy varies significantly across different tumor types and between individuals. Previous studies identified the tumor immune microenvironment (TIME) significantly influences therapeutic responses [11,12]. In this context, TIME was often classified as immune-positive or immune-escape, affecting immunotherapy efficacy.

Recent studies suggest that programmed cell death (PCD) extends beyond terminal cell fate and can regulate anti-tumor immunity [13]. Beyond traditional apoptosis, diverse PCDs, including pyroptosis, ferroptosis, necroptosis, and the newly defined cuproptosis and disulfidptosis, are found to modulate the TIME [14,15]. For instance, pyroptosis triggered the release of pro-inflammatory cytokines like interleukin-1 beta (IL-1β), which improved the immune response in BC models [16]. Ferroptosis was shown to enhance the sensitivity of RCC to immunotherapy by increasing lipid peroxidation-induced immunogenicity [6]. Furthermore, the concept of PANoptosis emerged to describe the molecular crosstalk among these pathways, suggesting that a synergistic activation of multiple death modes could release a broader spectrum of damage-associated molecular patterns and help address urological tumor heterogeneity and immune evasion [17]. While the individual roles of specific cell death modes have been extensively documented, a comprehensive synthesis of how multiple programmed cell death pathways collectively orchestrate the remodeling of the urological immune landscape is currently lacking. Understanding the interplay between these diverse death signals and immune cell infiltration may guide next-generation combination therapies.

Therefore, this review aimed to summarize the mechanisms of various PCDs in urological cancers, analyze their correlation with the immune microenvironment, and evaluate their potential as biomarkers or therapeutic targets to improve the efficiency of immunotherapy. By integrating these multi-dimensional insights, we provided a theoretical framework for future innovative drug development and precision oncology.

## 2 Methods

We conducted this narrative review using PubMed, Scopus, and Web of Science databases to identify studies that examined the regulatory networks, immune-microenvironmental effects, biomarker potential, and therapeutic interventions related to programmed cell death in urological cancers. We prioritized studies involving clinical, preclinical, and *in vitro* evidence on the interaction between programmed cell death pathways and the tumor immune microenvironment. Supplementary Table S1 provides a comparative overview of key molecular nodes, immune effects, representative agents, and translational implications is provided in Supplementary Table S1.

## 3 Ferroptosis: Iron-Dependent Lipid Peroxidation and Immune Remodeling

Ferroptosis is a form of regulated cell death driven by iron-dependent lipid peroxidation and subsequent plasma membrane rupture [18,19]. Mechanistically, it involves depletion of glutathione (GSH) and the inactivation of glutathione peroxidase 4 (*GPX4*), which leads to the lethal accumulation of reactive oxygen species (ROS) on cellular lipids [20,21]. Unlike apoptosis, ferroptosis can be immunogenic because it releases intracellular content sand inflammatory mediators.

### 3.1 Ferroptosis and PCa

#### 3.1.1 Ferroptosis Regulates PCa Immunity

In PCa, ferroptosis induction has been proposed to overcome apoptosis resistance in advanced disease. For instance, in LNCaP and PC3 cell lines, erastin inhibited system Xc−, whereas RAS-selective lethal 3 (RSL3) directly inhibited GPX4, and both treatments induced marked ferroptotic cell death [22]. In terms of immune regulation, the combination of icaritin and curcumol was found to inhibit PCa progression in RM-1 murine prostate cancer cells and DU145 RM-1 murine prostate cancer cells cell models by inducing both autophagy and ferroptosis, which subsequently modulated the gut microbiota/metabolism/immune axis, suppressed the *DNA methyltransferase 1 (DNMT1)/insulin-like growth factor-binding protein 2 (IGFBP2)/PD-L1* signaling pathway, and significantly increased the infiltration and activation of CD3^+^ CD8^+^ interferon-gamma (IFN-gamma)-producing T cells [23]. The N6-methyladenosine (m6A) reader YTH N6-methyladenosine RNA-binding protein F1 (YTHDF1) was also reported to promote immune evasion in PCa by stabilizing PD-L1 mRNA, thereby inhibiting both the cytotoxic activity of CD8^+^ T cells and T cell-mediated ferroptosis, thereby correlating with poor clinical prognosis [24]. In PC-3 and DU145 cells, the natural alkaloid evodiamine was shown to impair angiogenesis and induce ferroptosis by upregulating *Sema3A* and reducing *GPX4* expression, while simultaneously blocking lactate-induced histone lactylation and PD-L1 transcription, leading to PCa inhibition [25]. In castration-resistant prostate cancer (CRPC), the RNA-binding protein HnRNP L was identified as an immune-evasion driver that stabilized the *YY1*/PD-L1 axis, thereby inhibiting CD8^+^ T cell-mediated ferroptosis and reducing the efficacy of PD-1 blockade in PC-3 and DU145 cells [26]. The above results suggest that ferroptosis can activate the TIME of PCa to potentially improve immunotherapy efficacy.

#### 3.1.2 Modulating the Ferroptosis of PCa to Activate Anti-Tumor Immunity

Therefore, some studies have tried to manage PCa by targeting ferroptosis-associated immune regulation. For instance, researchers developed magnetic-targeted ZFPG nanoparticles that triggered a multienzyme cascade and sensitization to deplete glutathione and glucose, thereby inducing *HMOX1*-mediated immunogenic ferroptosis and remodeling the immunosuppressive tumor microenvironment in male mouse models [27]. Similarly, Cheng et al. [28] developed a biomimetic nanovesicle (FiFe@RBM) that inhibited the AKT-mTOR pathway and rewired lipid metabolism by increasing polyunsaturated fatty acids, thereby synergistically inducing apoptosis and ferroptosis to eradicate PC-3 tumors and suppress liver metastasis through the recruitment of natural killer (NK) cells in nude mice. Furthermore, in CRPC, novel p-PSMA-CAR-NK92MI cells were developed to specifically target prostate-specific membrane antigen-positive (PSMA+) C42 tumors, demonstrating superior tumoricidal efficacy both *in vitro* and *in vivo* [29]. Mechanistically, these engineered NK cells secreted high levels of IFN-gamma, which triggered ferroptosis as a primary pathway to eradicate cancer cells. Preclinical studies suggest that inducing ferroptosis through nanotechnology, natural compounds, or CAR-NK cells may help convert the “cold” microenvironment of PCa into a more immunologically active state. Future clinical translation will depend on identifying precise metabolic biomarkers to predict ferroptosis sensitivity and optimizing combinatorial therapies that trigger tumor-specific ferroptosis without compromising the effector functions of infiltrating immune cells.

### 3.2 Ferroptosis and BC

In BC, ferroptosis has been studied as a mechanism that may modulate the TIME and improve responses to anti-PD-L1 therapy. For instance, In BC tissues and cell lines, the m6A methyltransferase Wilms tumor 1-associated protein (WTAP) was found to be upregulated and associated with poor prognosis [30]. Mechanistically, *WTAP* installed m6A modifications on the 3′-untranslated region (3′-UTR) of *nuclear factor erythroid 2-related factor 2 (NRF2) mRNA*, which are subsequently recognized by the reader *YTHDF1* to enhance mRNA stability, thereby suppressing erastin-induced ferroptosis and promoting tumor cell growth. These findings support a potential immune-activating and antitumor role for ferroptosis in selected BC contexts. Consequently, several studies have developed novel nano- and ferroptosis-based therapies to manage BC via immune modulation. For example, in cisplatin- and tislelizumab-resistant BC patient-derived organoids (PDOs) and humanized patient-derived xenografts (PDX), the folate-targeted nanocomposite DIFP-FA integrated sonodynamic, chemodynamic, and chemotherapy to induce ferroptosis and immunogenic cell death, which effectively enhanced T lymphocyte infiltration and sensitized immune-tolerant tumors to anti-PD-1 therapy [31]. In orthotopic BC models, a novel intravesical nanoplatform (FBZ@BSA@PDA) was developed to trigger ferroptosis and immunogenic cell death (ICD) via GSH depletion and mitochondrial dysfunction, which effectively promoted dendritic cell maturation and T-cell activation to achieve synergistic anti-tumor efficacy [32]. Also in orthotopic BC models, a mannose-modified dual-responsive nanosystem (MPP@IKE-aPD-1/diABZI) was engineered to co-deliver the ferroptosis inducer IKE, a stimulator of interferon genes (STING) agonist, and anti-PD-1 antibodies, achieving a 94.5% tumor inhibition rate and a 92% reduction in lung metastasis by synchronizing ferroptosis-induced ICD with STING pathway activation to enhance CD8^+^ T cell infiltration and establish long-term immune memory [33]. In BC, Preclinical evidence suggests that ferroptosis may enhance the efficacy of PD-L1 blockade by releasing inflammatory signals that recruit effector T cells into the TIME. A translational priority is developing urological-specific delivery systems, including intravesical nanomedicines, that induce localized ferroptosis while limiting systemic toxicity and preserving urinary immune-cell viability.

### 3.3 Ferroptosis and RCC

#### 3.3.1 Ferroptosis Enhances RCC Immune Activation

RCC, particularly the clear cell subtype, has a distinctive dependence on antioxidant defense systems, making it potentially susceptible to ferroptosis compared to other urological malignancies [34,35]. In TIME, ferroptosis serves as a double-edged sword in RCC. On the one side, ferroptosis may support an immune-active TIME in RCC. For example, in RCC, the mitochondrial protein ferredoxin 1 (FDX1) functions as a dual regulator that initiated a pre-ferroptotic immune-alert state by triggering the cytosolic release of mitochondrial DNA (mtDNA) and mitochondrial double-stranded RNA (mt-dsRNA), which activated the cyclic GMP-AMP synthase (cGAS) and retinoic acid-inducible gene I/melanoma differentiation-associated protein 5 (RIG-I/MDA5) pathways to induce a robust type I interferon response and recruit CD8^+^ T cells [36].

In chromophobe RCC, immunogenomic profiling revealed an immune evasion microenvironment characterized by a profound depletion of CD8^+^ T cells and downregulated human leukocyte antigen (HLA) class Imolecules, suggesting that the enrichment of the ferroptosis pathway in these tumors may inform tailored strategies to improve tumor-specific T-cell infiltration and overcome resistance to current immunotherapies [37]. Similarly, *protein arginine methyltransferase 5* (*PRMT5*)*-mediated methylation of acyl-CoA synthetase long-chain family member 4* (*ACSL4*) at R549 promotes its proteasomal degradation to suppress lipid peroxidation, and targeting this axis sensitizes the tumor to anti-PD-1 therapy by combining ferroptotic cell death with immune-mediated tumor regression [6].

#### 3.3.2 Ferroptosis Induces RCC Immune Evasion

**Figure 1:**
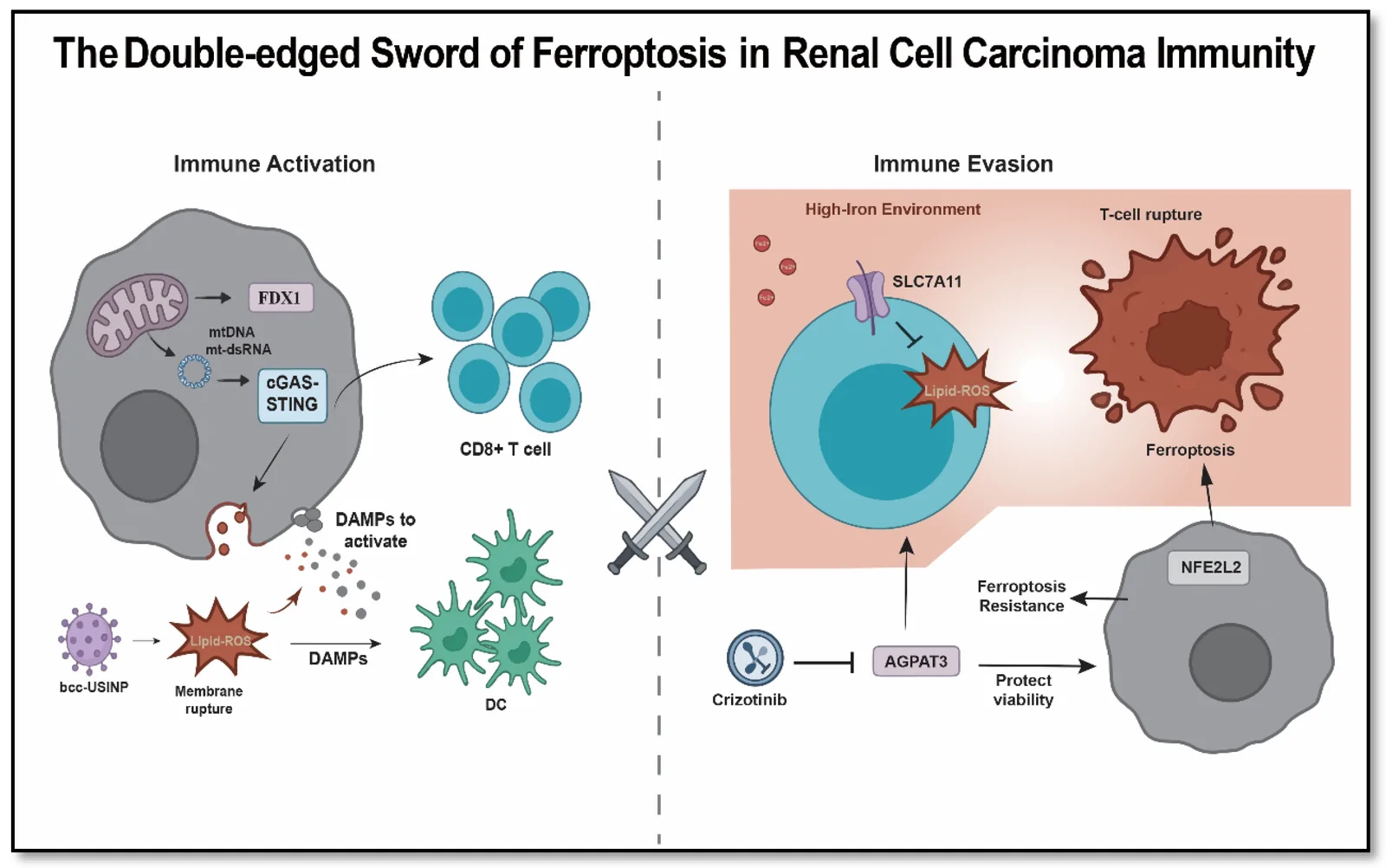
**The double-edged role of ferroptosis in renal cell carcinoma immunity**: In RCC, ferroptosis exerts dual effects on the tumor immune microenvironment. On one hand, ferroptosis inducers trigger mitochondrial DNA release, activate the cGAS-STING pathway, promote dendritic cell maturation, and enhance CD8^+^ T cell infiltration, thereby fostering anti-tumor immunity. On the other hand, ferroptosis resistance mechanisms or a high-iron environment could render infiltrating CD8^+^ T cells susceptible to ferroptotic death, leading to immune evasion. Pharmacological agents such as crizotinib may protect T cells by inhibiting ferroptosis. Abb: RCC, renal cell carcinoma; cGAS, cyclic GMP–AMP synthase; STING, stimulator of interferon genes; DAMP, damage-associated molecular pattern; DC, dendritic cell; SLC7A11, solute carrier family 7 member 11; NFE2L2, nuclear factor erythroid 2-like 2; AGPAT3, 1-acylglycerol-3-phosphate O-acyltransferase 3; CD8, cluster of differentiation 8.

On the other side, ferroptosis leads to immune evasion TIME. For instance, in individuals with sickle cell disease, disrupted 3D genome architecture in CD8^+^ T cells reduced the expression of solute carrier family 7 member 11 (SLC7A11) which renders the T cells themselves hypersensitive to ferroptosis, leading to premature immune cell death and a subsequent failure in anti-tumor immunity [38]. In renal medullary carcinoma, the loss of SWI/SNF-related matrix-associated actin-dependent regulator of chromatin subfamily B member 1 (SMARCB1) and the high-iron environment of sickle cell trait drive a transcriptional switch toward nuclear factor erythroid 2-like 2 (NFE2L2)-mediated ferroptosis resistance, allowing tumor-initiating cells to survive iron-induced oxidative stress while simultaneously triggering the ferroptotic depletion of infiltrating CD8^+^ T cells due to *SLC7A11* downregulation. Therefore, the clinical drug crizotinib and its enantiomer acted as potent ferroptosis inhibitors by targeting 1-acylglycerol-3-phosphate O-acyltransferase 3 (AGPAT3)-mediated PE-O-PUFA synthesis, thereby mitigating ischemia-reperfusion injury and enhancing anti-tumor immunity by preventing the immunosuppressive lipid-ROS accumulation in CD8^+^ T cells [39]. Furthermore, Liang et al. [40] developed a tumor-responsive bcc-USINPs, which leveraged an acidic-etched Fe^0^ core to catalyze potent ferroptosis and immunogenic cell death, which facilitated dendritic cell maturation and synergized with anti-PD-L1 therapy to establish a robust, long-term adaptive immune response. In RCC, evidence suggests that ferroptosis can influence the TIME in opposing directions, either supporting antitumor immunity or contributing to immune evasion. While targeted induction may recruit effector cells and support immune memory in selected models, certain genetic and clinical backgrounds may predispose immune cells to premature exhaustion. The translational value of ferroptosis-based immunotherapy will likely require a balance between tumor-specific oxidation and the preservation of immune cell viability. Future research should prioritize spatiotemporal delivery systems that selectively trigger ferroptosis within the tumor cells while utilizing metabolic “shields” to protect infiltrating immune cells from oxidative exhaustion. Biomarkers that distinguish patients likely to benefit from pro-ferroptotic induction from those at high risk for immune-cell toxicity will be important for tailoring ferroptosis-based immunotherapies across diverse RCC subtypes. Fig. 1 summarizes these findings.

These observations support a functional classification for ferroptosis-based interventions in RCC. In tumor-cell-dominant, ferroptosis-sensitive settings with preserved effector-cell fitness, selective induction of ferroptosis in tumor cells may be appropriate. Conversely, when infiltrating CD8^+^ T cells are ferroptosis-prone, as in iron-rich or sickle-cell-associated contexts, protection of immune cells from ferroptotic injury should take priority. Treatment selection should therefore integrate tumor-cell ferroptosis susceptibility, iron/lipid-redox status, and the abundance and functional state of infiltrating CD8^+^ T cells [6,35,36,39,40].

Although disulfidptosis is less developed than ferroptosis in urological oncology, it is directly relevant to the renal cancer immunotherapy context. Wang et al. reported that intermittent fasting combined with metformin induced disulfidptosis and enhanced the efficacy of anti-PD-1 therapy in renal cancer [15]. This study provides urological cancer-specific evidence linking disulfidptosis to immune-checkpoint response, although the evidence is still emerging and should be interpreted separately from non-urological disulfidptosis models.

## 4 Cuproptosis: A Mitochondrial Metabolic Trigger for Immune Remodeling

**Figure 2:**
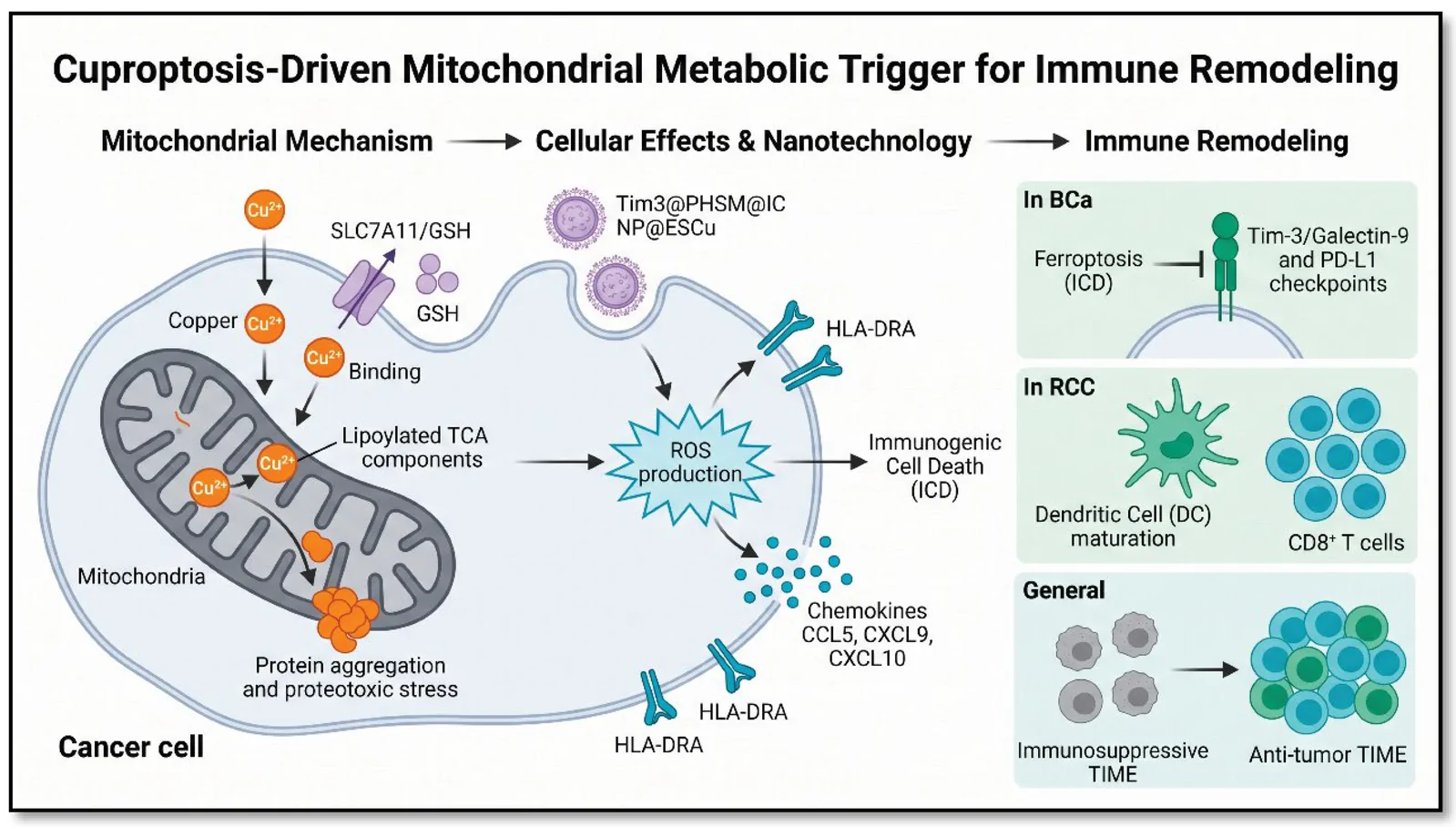
**Cuproptosis activated TIME via mitochondrial proteotoxic stress.** Mitochondrial Mechanism: Cu^2+^ bound to lipoylated TCA components, causing protein aggregation and mitochondrial dysfunction. This is regulated by SLC7A11 and GSH. Nano-intervention: Platforms like Tim3@PHSM@IC and NP@ESCu induced cuproptosis, increasing ROS and HLA-DRA expression, and stimulating ICD. Immune Effects: In reported BCa and RCC models, cuproptosis was associated with Tim-3/PD-L1 checkpoint modulation, DC maturation, and CD8^+^ T cell infiltration. Abb: TIME, tumor immune microenvironment; TCA, tricarboxylic acid cycle; HLA-DRA, major histocompatibility complex class II DR alpha; ICD, immunogenic cell death; PD-L1, programmed death-ligand 1; GSH, glutathione; BCa, bladder cancer; CD8, cluster of differentiation 8; DC, dendritic cell; RCC, renal cell carcinoma; ROS, reactive oxygen species; SLC7A11, solute carrier family 7 member 11; Tim-3, T-cell immunoglobulin and mucin domain 3. Tim3@PHSM@IC and NP@ESCu are the designations used for the reported nanoplatforms.

Cuproptosis, a recently described form of regulated cell death, results from the direct binding of copper to lipoylated components of the tricarboxylic acid (TCA) cycle, leading to proteotoxic stress and mitochondrial dysfunction [41,42]. *FDX1* is an upstream regulator of this process, contributing to the reduction of Cu^2+^ to the more reactive Cu^+^ and promoting protein lipoylation, thereby facilitating proteotoxic stress and mitochondrial dysfunction. In urological cancers, cuproptosis has been implicated in immune regulation and immunotherapy response. For instance, cuproptosis upregulated *major histocompatibility complex class II DR alpha (HLA-DRA) expression* via ROS production, and increased the secretion of *CCL5*, *CXCL9*, and *CXCL10* to promote T cell infiltration and synergized with anti-PD-1 therapy to suppress RCC progression [41]. In CRPC, *pyruvate dehydrogenase E1 subunit alpha 1 (PDHA1) induced* enzalutamide resistance by increasing *SLC7A11* expression and glutathione synthesis to chelate intracellular copper, thereby suppressing cuproptosis and establishing an immune-evasive metabolic phenotype [43]. These preclinical findings suggest that cuproptosis in cancer cells may restrain urological tumor progression and promote an anti-tumor TIME in selected contexts. Therefore, many researchers tried to improve immunotherapy efficacy via cuproptosis. For instance, in BC, the biomimetic Tim3@PHSM@IC nanoplatform integrated photothermal therapy with cuproptosis to induce robust immunogenic cell death, which overcome thermal resistance and competitively depressed the Tim-3/Galectin-9 checkpoint to reprogram the immunosuppressive TIME for enhanced immunotherapy [44]. In another BC study, ROS-responsive NP@ESCu nanoparticles leveraged the elesclomol-copper axis to trigger cuproptosis, which effectively reprogrammed the TIME and synergized with anti-PD-L1 therapy to enhance systemic anti-tumor immunity [45]. In RCC, the membrane-camouflaged mCGYL-LOx nanoplatform synergistically induced chemodynamic therapy and cuproptosis to stimulate immunogenic cell death, which promoted dendritic cell maturation and suppressed immune escape by downregulating PD-L1 through lactate consumption and finally resulted in cancer cell inhibition [46]. Cuproptosis may link metabolic stress to immune regulation in urological cancers, where it can induce proteotoxic tumor cell death and may counteract the immunosuppressive TIME by enhancing antigen presentation and promoting pro-inflammatory cytokine secretion. A key translational requirement is pairing cuproptosis-sensitivity biomarkers with delivery systems that control intracellular copper exposure, thereby enabling rational combination with ICIs. Fig. 2 exhibits above results.

## 5 Autophagy: A Metabolic Rheostat in Urological Immunotherapy

### 5.1 Autophagy Leads to an Immune Evasion TIME

Autophagy, a conserved lysosomal degradation pathway, plays a dual role in urological cancers by maintaining cellular homeostasis and modulating the TIME [47,48]. On one hand, autophagy can contribute to immune evasion and the progression of urological cancers. For example, in BC, the E3 ubiquitin ligase cullin 5 (CUL5) facilitated immune evasion by promoting the ubiquitination of polypyrimidine tract-binding protein 1 (PTBP1), which modulated the alternative splicing of rubicon autophagy regulator (RUBCN) to maintain autophagy and resist CD8^+^ T cell-mediated killing [49]. In another BC study, intracellular Bacillus Calmette-Guérin (BCG) infection resulted in immune subversion by inhibiting autophagic flux, which post-transcriptionally downregulated human leukocyte antigen class I (HLA-I) membrane expression to foster a myeloid-driven immunosuppressive TIME and predict poor clinical outcomes [50]. Similarly, in PCa, increased extracellular matrix stiffness activated the integrinbeta1/FAK/YAP axis to promote the expression of the deubiquitinase ubiquitin-specific peptidase 8 (USP8), which simultaneously stabilized PD-L1 and NBR1 autophagy cargo receptor (NBR1) [51]. Consequently, elevated *NBR1* (the autophagy receptor) triggered the selective autophagic degradation of major histocompatibility complex class I (MHC-I), thereby facilitating immune evasion and suggesting that *USP8* inhibition could sensitize high-Gleason score tumors to immunotherapy. In RCC, *circGRAMD4* promoted immune evasion by interacting with *RBM4* to stabilize *NBR1*, thereby impairing antigen presentation and inducing CD8^+^ T cell dysfunction [52]. These findings suggest that autophagy in urological cancer cells can support an immune-evasive TIME and contribute to poor immunotherapy response.

### 5.2 Autophagy Leads to an Immune Positive TIME

On the other hand, autophagy may support an anti-tumor TIME [53]. For instance, in a PCa study, the C-X-C motif chemokine ligand 12/C-X-C motif chemokine receptor 4 (CXCL12/CXCR4) axis activated CCAAT/enhancer-binding protein beta (C/EBP-beta) to deregulate autophagy and impair mitochondrial polarization in NK cells, leading to their metabolic dysfunction and immune exhaustion [54]. These findings suggest that autophagy in NK cells can support antitumor function.

**Figure 3:**
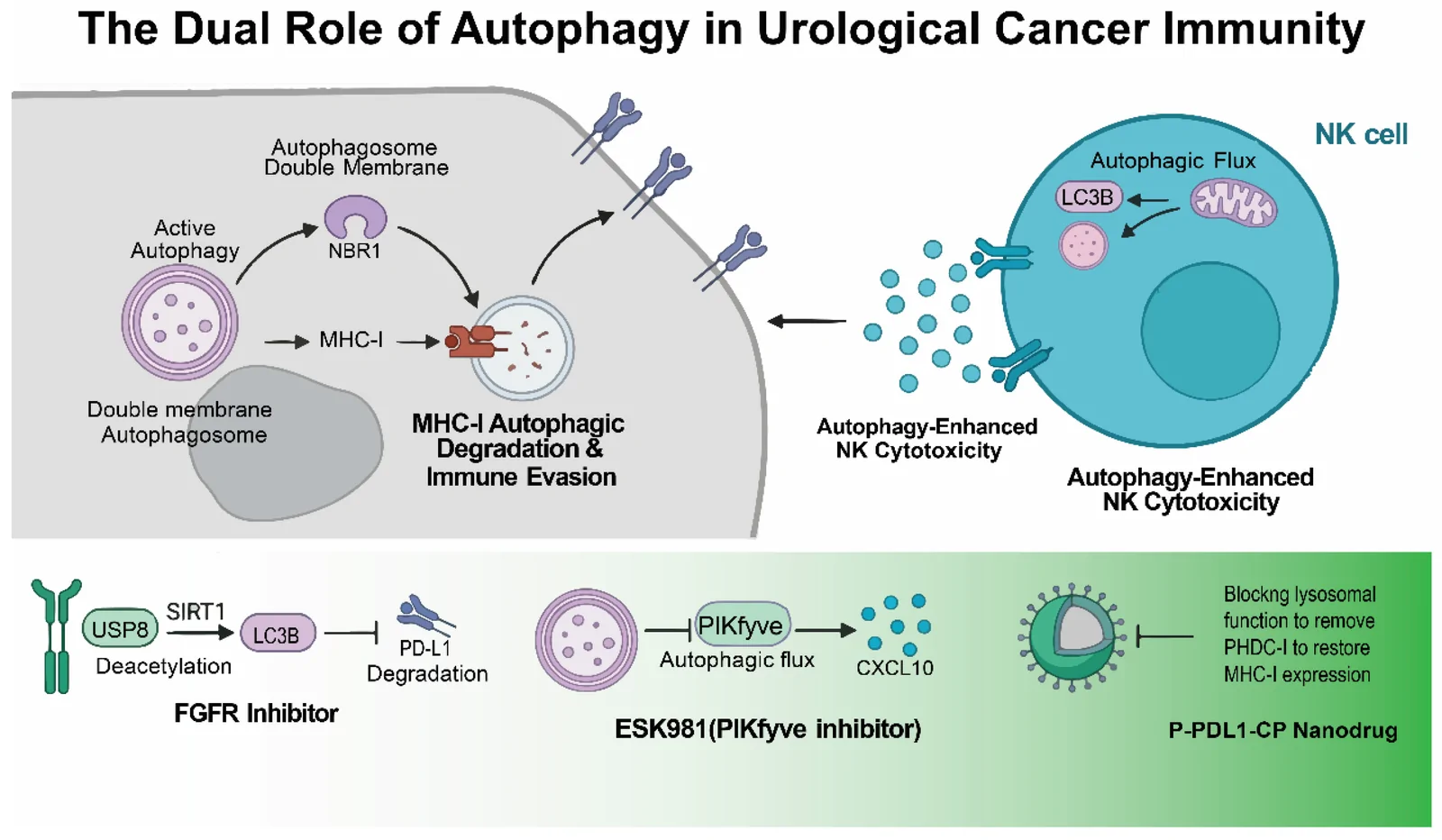
**The dual role of autophagy in urological cancer immunity**: Autophagy plays a context-dependent role in urological cancers. In tumor cells, active autophagy can promote immune evasion by selectively degrading MHC-I via the NBR1 pathway and stabilizing PD-L1. Conversely, in NK cells, autophagy can enhance cytotoxicity and metabolic fitness. Pharmacological interventions, including FGFR inhibitors, ESK981 (PIKfyve inhibitor), and P-PDL1-CP nanodrug, can modulate autophagic flux to restore MHC-I expression, degrade PD-L1, or promote CXCL10-mediated T cell infiltration, thereby potentially reducing immune evasion and improving immunotherapy efficacy in selected settings. Abb: MHC-I, major histocompatibility complex class I; NBR1, NBR1 autophagy cargo receptor; NK, natural killer cell; FGFR, fibroblast growth factor receptor; CXCL10, C-X-C motif chemokine ligand 10; PD-L1, programmed death-ligand 1; PIKfyve, phosphoinositide kinase, FYVE-type zinc finger containing. ESK981 and P-PDL1-CP are the designations used for the reported agent and nanoplatform, respectively.

Many potential treatments are developing by targeting autophagy [55]. For instance, fibroblast growth factor receptor (FGFR) inhibitors suppressed IFN-gamma-induced PD-L1 expression by restoring *sirtuin 1 (SIRT1) levels* to promote *microtubule-associated protein 1 light chain 3 beta (LC3B) deacetylation* and subsequent autophagy-lysosomal degradation, thereby reversing T cell suppression and enhancing the efficacy of immune checkpoint blockade in *FGFR3*-altered BC [56]. In RCC, the APm/Ce6/HIF nanoplatform synergized with ultrasound to overcome SDT-induced hypoxia and upregulated autophagy, thereby inhibiting HIF-2alpha-mediated resistance and activating anti-tumor immune activity to enhance the efficacy of immune checkpoint blockade [57]. In another RCC study, the orally bioavailable multi-tyrosine kinase inhibitor ESK981 targeted the lipid kinase phosphoinositide kinase, FYVE-type zinc finger containing (PIKfyve) to inhibit autophagic flux, which upregulated *CXCL10* via the interferon-gamma pathway to promote functional T cell infiltration, effectively enhancing the therapeutic response to immune checkpoint blockade [58]. In PCa, the combination of icaritin and curcumol inhibited tumor progression by modulating the gut microbiota and increasing short-chain fatty acids, which downregulated the *DNMT1*/*IGFBP2*/PD-L1 axis to suppress autophagy and enhance CD8^+^ T cell-mediated anti-tumor immunity [23]. In another PCa study, the pH-sensitive P-PDL1-CP nanodrug utilized a PDPA core to disrupt lysosomal function and block autophagic flux, thereby preventing the degradation of MHC-I and increasing tumor vulnerability to tumor necrosis factor alpha (TNF-alpha) and cytotoxic T lymphocyte (CTL)-mediated killing [59]. Moreover, this dual-action platform synergized with released anti-PD-L1 to promote dendritic cell maturation and a robust anti-tumor immune memory response. Autophagy in cancer cells can induce an immune-evasive microenvironment and impair the efficacy of immunotherapy. However, in immune cells such as NK cells, autophagy can enhance their anti-cancer capabilities. Accordingly, autophagy-targeted interventions should be classified by the principal target cell. In cancer cells in which autophagy drives MHC-I loss, PD-L1 stabilization, or resistance to CD8^+^ T cell-mediated killing, tumor-cell-selective autophagy inhibition may be beneficial. Conversely, when autophagic flux is impaired in NK cells, restoring autophagy may preserve metabolic fitness and cytotoxicity. These approaches should be selected according to the target cell type, because systemic autophagy inhibition could compromise NK-cell function [50,51,52,53,54,55,56]. This functional dichotomy complicates for developing effective therapeutic strategies. Accordingly, autophagy should be modulated in a cell-type-specific manner rather than systemically. Fig. 3 summarizes this regulatory network.

## 6 Pyroptosis: An Inflammatory Engine for Tumor Immune Modulation

### 6.1 Pyroptosis Promotes Immune Activation

Pyroptosis, a gasdermin-mediated inflammatory form of programmed cell death, involves cell swelling and plasma membrane rupture, and the massive release of pro-inflammatory cytokines such as IL-1β and interleukin-18 (IL-18) [60,61]. Pyroptosis may influence immune regulation and immunotherapy response in urological cancers. For instance, in RCC, the aberrant upregulation of STING served as a tumor-promoting mechanism by suppressing endoplasmic reticulum (ER) stress-mediated and *gasdermin D (GSDMD)-dependent* pyroptosis, whereas its pharmacological degradation via a proteolysis-targeting chimera (PROTAC) triggers an inflammatory programmed cell death that effectively recruited CD4^+^ and CD8^+^ T cells and restored antitumor immunity [62]. In PCa, docetaxel chemotherapy overcome *SKP2*-mediated degradation of gasdermin E (GSDME) by inhibiting the AKT pathway, thereby triggering pyroptosis to recruit CD8^+^ T cells and NK cells, which synergized with avelumab to enhance antitumor immunity [63]. In another PCa study, the E3 ligase cell division cycle 20 (CDC20) negatively regulated the TIME by targeting *GSDME* for ubiquitination-mediated degradation, whereas pharmacological inhibition of *CDC20* via Apcin restored *GSDME*-dependent pyroptosis and prooted CD8^+^ T cell infiltration to synergize with anti-PD-1 therapy [64]. In BC, mannose functioned as a metabolic-epigenetic modulator by directly binding to pyruvate kinase M2 (PKM2) to inhibit its enzymatic activity, which activated *PKM2* nuclear translocation and subsequent *NLR family pyrin domain containing 1 (NLRP1)/caspase-1/GSDMD-dependent* pyroptosis to activate robust antitumor immunity [16]. Preclinical findings suggest that pyroptosis may suppress RCC, PCa, and BC progression by promoting a more immune-active TIME.

### 6.2 Targeting Pyroptosis to Suppress Cancer Progression

Many potential novel treatments were developed based on the above evidence. For instance, in RCC, the CD44-targeted PDA-Mn-HA nanoparticles activated the ROS-STING-p38/mitogen-activated protein kinase (MAPK) pathway to drive M1 macrophage polarization and trigger *GSDME*-dependent pyroptosis, effectively remodeling the immune microenvironment to suppress RCC growth and metastasis [65]. In PCa, the MIT@ZIF-8 nanoplatform enhance an immune-positive TIME for PCa by amplifying mitoxantrone-induced pyroptosis and immunogenic cell death, which recruited cytotoxic CD8^+^ T cells and depleted Treg cells to sensitize refractory tumors to anti-CTLA-4 immunotherapy [66]. Similarly, the genetically engineered PSMAscFv-EVN-*GSDMD* extracellular vesicle selectively promoted the N-terminal domain of *GSDMD* to PSMA-positive cells to stimulate targeted pyroptosis, effectively converting the immunosuppressive “cold” tumor microenvironment into an immunogenic “hot” one to suppress tumor growth [67]. In BC, the microwave-sensitized Mn-ZrMOF@DAC nanoplatform induced *GSDME*-mediated pyroptosis and oxidative damage, which transported weak immunostimulatory signals into a robust systemic immune response that synergized with anti-PD-1 therapy to overcome the immunosuppressive TIME [68]. Furthermore, the engineered MINS@MΦ macrophage delivery system utilized BCG-induced inflammation for targeted recruitment and triggered light-responsive NIG-mediated self-pyroptosis to release CpG and iron ions, which effectively polarized tumor-associated macrophages toward an M1 phenotype to enhance the efficacy of adjuvant immunotherapy without obvious systemic toxicity in that model [69]. For pyroptosis-inducing therapies, central translational barriers include the absence of dedicated clinical trials, the need to confine inflammatory damage to tumors, and the need for predictive biomarkers of response and membrane-repair-mediated resistance.

## 7 PANoptosis: A Multi-Pathway Strategy to Address Tumor Heterogeneity

### 7.1 PANoptosis Activates Anti-Tumor Immunity

PANoptosis, an integrated inflammatory programmed cell death pathway co-regulated by pyroptosis, apoptosis, and necroptosis, may act as an integrative regulator of the TIME in urological cancers. The evidence reviewed above indicates that pyroptosis may restrain urological cancer progression by promoting an immune-active TIME and may improve immunotherapy efficacy in selected models. Necroptosis, a regulated form of necrotic cell death mediated by the *RIPK1*/*RIPK3*/*MLKL* signaling axis, can eliminate urological cancer cells that have developed resistance to apoptosis [70]. In PCa, the MSCN cryo-nanocatalyst significantly enhanced cryo-cytotoxicity by elevating the freezing point to trigger osmotic-related necroptosis, which upregulated PD-L1 and stimulated systemic antitumor memory immune responses when synergized with localized anti-PD-L1 blockade [71].

### 7.2 PANoptosis Based Therapy Development

Similarly, PANoptosis has shown anti-tumor potential in preclinical urological cancer models. For instance, in BC, ultrasound-responsive Mn/Se nanozymes synergized with PD-1 blockade by activating the STING pathway and PANoptosis, which triggered potent immunogenic cell death to remodel the TIME, promoted dendritic cell maturation, and expanded systemic CD8^+^ T-cell immunity and was associated with tumor inhibition in the reported model [14]. In PCa, the biomimetic absent in melanoma 2 (AIM2)-activator implant (Fe_3_O_4_/AIPH/DDP@PLGA) triggered nuclear and mitochondrial DNA dual-damage to initiate PANoptosome assembly, effectively converting immune evasion into a positive TIME by inducing PANoptosis and robust CD8^+^ T-cell recruitment [17]. These findings suggest that orchestrating PANoptosis may be a strategy for enhancing the efficacy of immune checkpoint blockade in heterogeneous urological cancers. The central translational question is whether PANoptotic signaling can be selectively amplified in malignant cells while preserving infiltrating immune-cell function. Together, these findings suggest that PANoptosis may provide a framework for immune modulation in urological cancer, although clinical validation remains limited.

**Figure 4:**
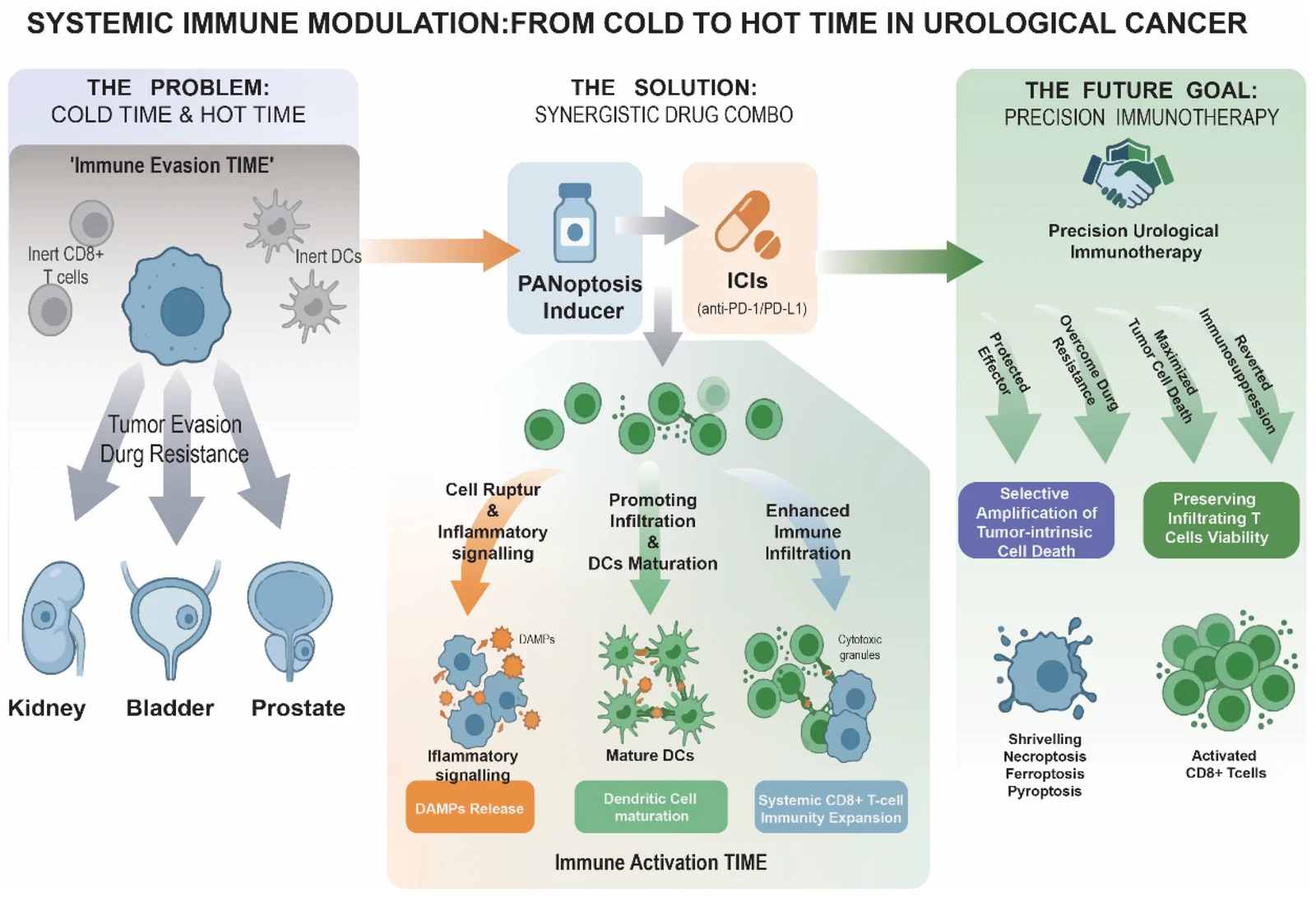
**Systemic immune modulation: from cold to hot TIME in urological cancer.** The left panel illustrates the “cold” immune-evasion TIME characterized by inert CD8^+^ T cells and dendritic cells, contributing to tumor evasion and drug resistance. The right panel presents a synergistic strategy combining PANoptosis inducers with immune checkpoint inhibitors. This approach triggered multiple programmed cell death pathways (necroptosis, ferroptosis, pyroptosis), released damage-associated molecular patterns (DAMPs), promoted dendritic cell maturation, and expanded systemic CD8^+^ T cell immunity. Selective amplification of tumor-intrinsic cell death while preserving infiltrating T cell viability may help convert the “cold” TIME into a more immune-activated TIME. Abbreviations: CD8, cluster of differentiation 8; DAMP, damage-associated molecular pattern; ICI, immune checkpoint inhibitor; TIME, tumor immune microenvironment.

Furthermore, emerging evidence suggests that certain therapeutic interventions can simultaneously trigger multiple programmed cell death pathways. For instance, in RCC, the biomimetic Cu_2_O-OMV nanozyme induced a synergistic cuproptosis-pyroptosis cell death program that amplified immunogenic cell death and CTL infiltration, thereby enhancing tumor immunogenicity and the efficacy of anti-PD-L1 therapy and was associated with reduced metastasis [72]. In BC, the dual-organelle-targeted aggregation-induced emission (AIE) photosensitizer DY disrupted lipid homeostasis in the ER and lipid droplets to initiate a synergistic cascade of ferroptosis and pyroptosis, which triggered robust immunogenic cell death and enhanced the efficacy of photodynamic therapy [73]. Targeting synergistic programmed cell death modes is emerging as an active area of research. Its multi-target and multi-pathway characteristics may help address resistance to cancer immunotherapy. However, the inherent complexity of such multi-modal interventions necessitates the development of robust and sophisticated analytical tools to further explore their therapeutic potential. Fig. 4 summarizes these findings.

## 8 Future Perspectives

### 8.1 Current Advances and Disadvantages

PCD provides a useful framework for understanding how urological tumors interact with the TIME. These pathways do not act in isolation. Ferroptosis, autophagy, pyroptosis, and PANoptosis may ferroptosis, autophagy, pyroptosis, and PANoptosis may influence antigen presentation, immune-cell recruitment, checkpoint sensitivity, and treatment resistance. At the same time, the dual roles of ferroptosis and autophagy emphasize that translational benefit will depend on cell-type-specific control rather than indiscriminate pathway activation. There are also important practical barriers. Most supporting data come from cell lines, nude mice, xenografts, orthotopic models, patient-derived xenografts, or proof-of-concept nanoplatform studies, whereas clinically annotated patient cohorts remain comparatively limited. This gap means that claims regarding prognostic biomarkers, treatment sensitivity, and toxicity management still require more direct validation in human disease settings.

### 8.2 Novel Techniques to Advance the Field

First, the investigation of programmed cell death in urological cancers is advancing through integrative research methodologies, including artificial intelligence (AI), spatial transcriptomics, and multi-omics mass spectrometry [74,75]. For instance, AI can predict protein structures and their potential function aldomains [76]. Simultaneously, spatial transcriptomics and single-cell technologies allow the study and validation of post-translational modifications across diverse cellular states and functional contexts [77]. Furthermore, natural products and their derivatives have increasingly been investigated in urological cancers as modulators of cell death-related and therapeutic signaling pathways [78]. For instance, the combination of icaritin and curcumol was found to inhibit PCa progression in RM-1 and DU145 cell models by inducing both autophagy and ferroptosis, which subsequently modulated the gut microbiota/metabolism/immune axis, suppressed the *DNMT1*/*IGFBP2*/PD-L1 signaling pathway, and significantly increased the infiltration and activation of CD3^+^ CD8^+^IFN-gamma T cells [23]. Multi-omics mass spectrometry may identify candidate active components and their primary target proteins, thereby elucidating the molecular mechanisms underlying these therapeutic effects [79,80].

Moreover, Spatial transcriptomics provides a “snapshot” of the tumor, future research should prioritize the integration of clinically annotated patient data with liquid biopsy approaches for programmed cell death-related circulating biomarkers [81,82]. This would allow clinicians to monitor the PCD-driven transition of the TIME in real-time, improve patient stratification, and dynamically adjust immunotherapy regimens before clinical resistance manifests. To address the autophagy paradox and other PCD-related contradictions, the development of smart nanocarriers or engineered exosomes may be important [83,84]. These platforms can be designed to respond to the specific acidic or hypoxic microenvironment of BC or PCa, ensuring that programmed cell death-inducing agents are selectively delivered to malignant cells while reducing off-target toxicity and shielding infiltrating effector T cells and NK cells from unintended collateral damage. In parallel, PDX models, orthotopic systems, and emerging cellular technologies should be used to distinguish proof-of-concept activity from strategies with realistic translational potential. For disulfidptosis specifically, the most directly relevant evidence in this review comes from RCC, in which intermittent fasting combined with metformin was reported to induce disulfidptosis and enhance anti-PD-1 efficacy [16]. Studies in hepatocellular carcinoma or generic hypoxic-tumor systems may provide mechanistic background, but they should not be used as primary evidence for urological cancer [85,86]. A practical framework is to classify PCD interventions according to the intended target cell and its immune context: (i) selectively induce tumor-cell PCD when it is expected to generate immunogenic signals while effector cells are preserved; (ii) suppress or buffer PCD in vulnerable immune cells, such as ferroptosis-prone CD8^+^ T cells; and (iii) use compartment-selective delivery when the same pathway has opposing effects in malignant and immune cells [39,40,50,51,52,53,54,55,56].

Clinical translation should proceed with caution. For PCD inducers, pharmacodynamic biomarkers should demonstrate pathway engagement in tumor cells without excessive injury to immune or normal tissues; the balance between tumor-cell death and preserved immune-cell function should be evaluated in clinically relevant models. Nanoplatforms further require evidence of reproducible manufacture, biodistribution, tumor penetration, clearance, and safety across heterogeneous patient populations [87,88].

## 9 Conclusion

The interplay between diverse programmed cell death pathways and the TIME may shape therapeutic opportunities in urological cancers. Future advances will depend on mechanistic rigor, clinically relevant patient stratification, biomarker-guided intervention, toxicity-aware precision delivery strategies, and a clearer bridge between preclinical proof-of-concept systems and patient-directed application.

## Data Availability

The data that support the findings of this study are available from the corresponding author upon reasonable request.

## Supplementary Materials

The supplementary material is available online at https://www.techscience.com/doi/10.32604/or.2026.087565/s1.

## Abbreviations

ACSL4	Acyl-CoA synthetase long-chain family member 4
AI	Artificial intelligence
AIM2	Absent in melanoma 2
BC	Bladder cancer
BCG	Bacillus Calmette-Guérin
CAR-NK	Chimeric antigen receptor-engineered natural killer cell
ccRCC	Clear cell renal cell carcinoma
CRPC	Castration-resistant prostate cancer
CTL	Cytotoxic T lymphocyte
CTLA-4	Cytotoxic T-lymphocyte-associated protein 4
DAMP	Damage-associated molecular pattern
DNMT1	DNA methyltransferase 1
FDX1	Ferredoxin 1
FGFR	Fibroblast growth factor receptor
GPX4	Glutathione peroxidase 4
GSH	Glutathione
GSDMD	Gasdermin D
GSDME	Gasdermin E
HLA	Human leukocyte antigen
HLA-DRA	Major histocompatibility complex class II DR alpha
HLA-I	Human leukocyte antigen class I
ICD	Immunogenic cell death
ICI	Immune checkpoint inhibitor
IFN	Interferon
IGFBP2	Insulin-like growth factor-binding protein 2
IL	Interleukin
MHC-I	Major histocompatibility complex class I
NK cell	Natural killer cell
NRF2	Nuclear factor erythroid 2-related factor 2
PCa	Prostate cancer
PCD	Programmed cell death
PD-1	Programmed cell death protein 1
PD-L1	Programmed death-ligand 1
PDHA1	Pyruvate dehydrogenase E1 subunit alpha 1
PDO	Patient-derived organoid
PDX	Patient-derived xenograft
PBRM1	Polybromo 1
PIKfyve	Phosphoinositide kinase, FYVE-type zinc finger containing
PRMT5	Protein arginine methyltransferase 5
PROTAC	Proteolysis-targeting chimera
PSMA	Prostate-specific membrane antigen
RCC	Renal cell carcinoma
ROS	Reactive oxygen species
RSL3	RAS-selective lethal 3
SLC7A11	Solute carrier family 7 member 11
STING	Stimulator of interferon genes
TCA	Tricarboxylic acid
TIME	Tumor immune microenvironment
TNF	Tumor necrosis factor
UC	Urothelial carcinoma
USP8	Ubiquitin-specific peptidase 8
WTAP	Wilms tumor 1-associated protein
YTHDF1	YTH N6-methyladenosine RNA-binding protein F1
