# Supplementary material for: Programmed Cell Death in Urological Cancers: Orchestrating the Immune Microenvironment and Immunotherapy
Source: Oncol Res. 2026 Sep 14;34(10):11. doi: 10.32604/or.2026.087565 (PMC13591326; doi:10.32604/or.2026.087565)
Supplement: Supplementary file 1 [file OncolRes-34-87565-s001.zip › TSP_OR_87565-s001.docx]

**Supplementary Table S1:** Functional summary of programmed cell death pathways.

| **Pathway** | **Cancer Type** | **Key Driver/Example** | **Predominant Immune Effect** | **Therapeutic Implication** | **Representative Refs.** |
| --- | --- | --- | --- | --- | --- |
| Ferroptosis | PCa | GPX4 inhibition, YTHDF1/PD-L1, CAR-NK | Can enhance CD8+ T-cell activity and immunogenicity | Tumor-selective induction may sensitize immunotherapy | [20–27] |
| Ferroptosis | BC | WTAP/NRF2, intravesical ferroptosis nanoplatforms | Promotes ICD, dendritic-cell maturation, and T-cell infiltration | Localized induction may overcome checkpoint resistance | [28–31] |
| Ferroptosis | RCC | FDX1, PRMT5/ACSL4, SLC7A11 vulnerability in T cells | Dual effect: tumor killing but possible immune-cell ferroptosis | Requires biomarker-guided, cell-type-specific control | [6,33–37] |
| Cuproptosis | RCC, PCa, BC | FDX1-related copper toxicity, PDHA1/SLC7A11, nanoplatforms | May enhance antigen presentation and T-cell recruitment | Potential adjunct to checkpoint blockade; evidence remains preclinical | [39–44] |
| Autophagy | RCC, BC, PCa | PBRM1, USP8, PIKfyve, PD-L1/MHC-I regulation | Tumor-cell autophagy often promotes immune evasion; immune-cell autophagy may be protective | Favors compartment-specific modulation rather than global inhibition | [21,46–55] |
| Pyroptosis/PANoptosis | RCC, PCa, BC | GSDMD/GSDME, STING, AIM2, nano-immunotherapy | Promotes inflammatory signaling, DAMP release, and T-cell recruitment | Promising for immune activation, but safety and translational control are critical | [13,15,16,58–69] |

Notes: Abbreviations: ACSL4, acyl-CoA synthetase long-chain family member 4; AIM2, absent in melanoma 2; BC, bladder cancer; CAR-NK, chimeric antigen receptor-engineered natural killer cell; CD8, cluster of differentiation 8; DAMP, damage-associated molecular pattern; FDX1, ferredoxin 1; GPX4, glutathione peroxidase 4; GSDMD, gasdermin D; GSDME, gasdermin E; ICD, immunogenic cell death; MHC-I, major histocompatibility complex class I; NRF2, nuclear factor erythroid 2-related factor 2; PBRM1, polybromo 1; PCa, prostate cancer; PD-1, programmed cell death protein 1; PD-L1, programmed death-ligand 1; PDHA1, pyruvate dehydrogenase E1 subunit alpha 1; PIKfyve, phosphoinositide kinase, FYVE-type zinc finger containing; PRMT5, protein arginine methyltransferase 5; RCC, renal cell carcinoma; SLC7A11, solute carrier family 7 member 11; STING, stimulator of interferon genes; USP8, ubiquitin-specific peptidase 8; WTAP, Wilms tumor 1-associated protein; YTHDF1, YTH N6-methyladenosine RNA-binding protein F1.
